# Transcriptomic datasets of Verticillium wilt resistant and non-resistant *Gossypium barbadense* varieties during pathogen inoculation

**DOI:** 10.1038/s41597-023-02852-2

**Published:** 2024-01-02

**Authors:** Xianpeng Xiong, Cong Sun, Bin Chen, Jie Sun, Cong Fei, Fei Xue

**Affiliations:** 1 Shenzhen Branch, Guangdong Laboratory of Lingnan Modern Agriculture, Genome Analysis Laboratory of the Ministry of Agriculture and Rural Affairs, Agricultural Genomics Institute at Shenzhen, Chinese Academy of Agricultural Sciences, Shenzhen, 518120 China; 2 State Key Laboratory of Cotton Biology, Institute of Cotton Research, Chinese Academy of Agricultural Sciences, Anyang, 455000 China; 3 Key Laboratory of Oasis Eco-Agriculture, College of Agriculture, Shihezi University, Shihezi, 832000 China; 4 Department of Life Sciences, Yuncheng University, Yuncheng, 044000 China

**Keywords:** Plant stress responses, Plant genetics

## Abstract

Cotton is a significant cash crop and the primary source of natural fiber globally. Among the numerous diseases encountered in cotton production, Verticillium wilt is one of the most serious, caused by the pathogen *Verticillium dahliae* (*V. dahliae*). Unfortunately, there are no effective targeted methods to combat this disease. Genomic resources for Verticillium wilt resistance primarily exist in *Gossypium barbadense* (*G. barbadense*). Regrettably, there have been limited transcriptomic comparisons between *V. dahliae*-resistant and -susceptible varieties of *G. barbadense* due to the scarcity of susceptible resources. In this study, we conducted a transcriptome analysis on both *V. dahliae*-resistant and -susceptible varieties of *G. barbadense* at the 0, 12, 24 and 48 hours after *V. dahliae* inoculation. This comparative transcriptome analysis yielded high-quality data and offered new insights into the molecular mechanisms underlying cotton’s resistance against this destructive pathogen.

## Background & Summary

Cotton, as one of the prominent fiber crops, serves as vital renewable natural fiber source, which contributes to approximately 35% of global fiber production^1,2^. In 2022, China becomes the second-largest cotton-producing country worldwide, yielding a total production of 5.977 million tons^3^. However, various diseases cause the loss of cotton production, with cotton Verticillium wilt (CVW) emerging as one of the most widespread and great threat to cotton production^4^. Over 40% of the cotton planting area in China being affected by *V. dahliae*, and this disease has become a main threat to the sustainable development of cotton production^5^.

CVW is a soil-borne vascular fungal disease, which is primarily caused by the pathogen of *V. dahliae*^6,7^. Root exudates induces *V. dahliae* germinaion and *V. dahliae* initiates invasion of the host plant through the root system. The pathogen then penetrates the root epidermal gaps via the root tip or natural wounds, ultimately growing vertically into the root vascular tissue. Subsequently, the spores of *V. dahliae* rapidly spreads to the cotton stems and leaves, resulting in gradual wilting and potential death of the cotton plant^8,9^. The microsclerotia of *V. dahliae* can survive up to 10 years, coupled with its wide range of hosts and the lack of effective fungicides for prevention or eradication, poses significant challenges in controlling the disease^5^. Consequently, the most effective, economical, safe, and environmentally friendly integrated disease management approach for controlling Verticillium wilt is planting and breeding resistant cultivars.

*Gossypium barbadense* (*G. barbadense*) and *Gossypium hirsutum* (*G. hirsutum*) are two major cultivated allopolyploid species, originating from natural hybridization and polyploidization events between diploid A0 and D5 genomes approximately 1–2 million years ago (MYA)^10^. These two cotton species exhibit significant differences in traits, such as stress tolerance, fiber quality, and lint yield^11^. These variations can be attributed to different processes of natural selection and human domestication. *G. hirsutum* is extensively cultivated due to its superior yield performance and better adaptability, contributing to over 90% of the global cotton fiber production^12,13^. *V.dahilae* impacts more than half of the cotton acreage, and *G. hirsutum* is notably susceptible to *V. dahliae*. This susceptibility leads to a substantial reduction in both fiber quality and yield^14^. Notably, *G. barbadense* demonstrates high resistance or immunity to cotton *V. dahliae*, making it a valuable resource for identifying key genes associated with Verticillium wilt resistance and uncovering the corresponding regulatory mechanisms^15,16^. However, the lack of *V. dahliae*-susceptible *G. barbadense* germplasm resources has limited the research focus to comparing *V. dahliae-*resistant *G. barbadense* with either *V. dahliae*-resistant *G. hirsutum* or *V. dahliae*-susceptible *G. hirsutum* varieties^17–22^. Currently, there is only one study comparing transcriptomic differences between *V. dahliae*-resistant and -susceptible varieties of *G. barbadense*, but the transcriptome data has not been released^16^. The rare bioinformatic data limited the deep understanding of how immune network of *G. barbadense* play role in defeating the invasion of *V. dahliae*.

In this study, we performed a comparison of the dynamic changes in the transcriptomes of resistant and susceptible *G. barbadense* varieties before and after inoculation with *V. dahliae* using RNA-seq. Our study demonstrates that the disease-resistant verity exhibits higher expression levels of genes involved in the suberin biosynthetic process, phenylpropanoid biosynthetic process, and other biological pathways associated with the establishment of cell physical barriers under normal growth conditions. Furthermore, we observed that the transcriptome response of the resistant variety is less sensitive even after invasion by Verticillium wilt, suggesting that its stronger physical barrier may play a significant role in conferring resistance against *V. dahliae*.

## Methods

### Plant material

Two *Gossypium barbadense* varieties, namely *V. dahliae*-resistant *G. barbadense* ‘Xinhai 47’(BR) and the *V. dahliae*-susceptible *G. barbadense* ‘Shidahaigan 1’ (BS) were selected for pathogenicity assays and RNA-seq analysis. To facilitate proper growth of the seedlings, the germinated seeds were incubated in controlled conditions in an incubator with a photoperiod of 16 hours of light and 8 hours of darkness; maintaining day and night temperatures of 25 °C and 23 °C respectively.

### Disease resistance identification

**Fig. 1:**
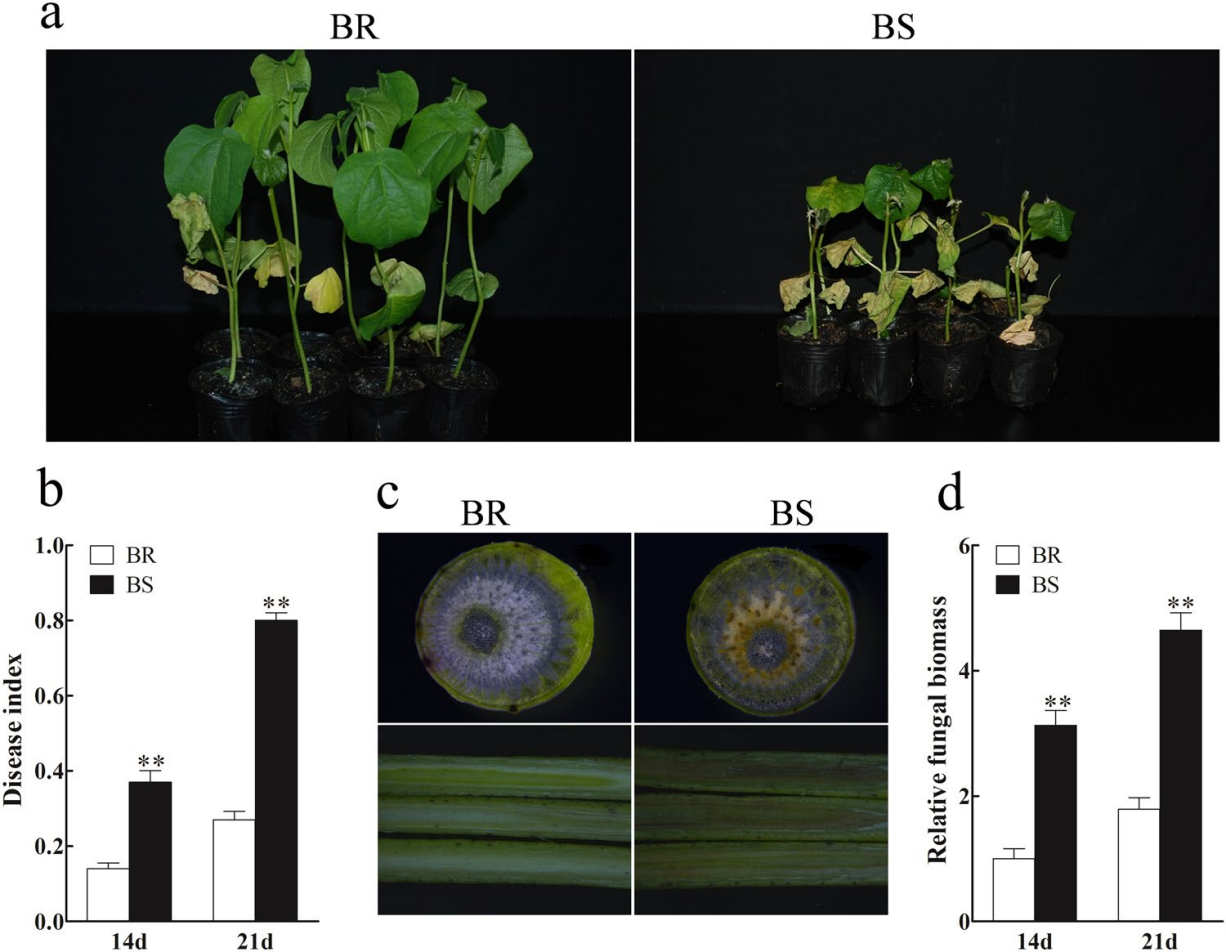
Comparison of *Verticillium dahliae* (*V. dahliae*) resistance. (**a**) The phenotype of BR and BS at 14 days post-infection (dpi) with *V. dahliae*, visual observations were made. (**b**) The disease index of both varieties was assessed at 14 and 21 dpi. (**c**) The phenotype of stems from BR and BS plants was examined 14 days after inoculation with *V. dahliae*. (**d**) The relative fungal biomass in the stems of BR and BS at 14 days post-inoculation (dpi) was determined. All data presented are expressed as mean ± standard deviation based on three independent biological replicates. Statistical analysis was performed using the T-test (P < 0.05, *P < 0.01).

Two-leaf-stage cotton seedlings were infected with the highly aggressive defoliating *V. dahliae* strain “V991” by using the root irrigation method^23^.The strain was kindly provided by Associate Professor Yanfei Sun (Shihezi University). The concentration of “V991” spore liquid was adjusted to 106 spores/mL by using a hemocytometer, followed by the application of 20 mL per plant into the nutrient bowls. Subsequently, the plants were incubated for further cultivation. At 14 days post-infection (dpi), the BR plants exhibited mild symptoms of Verticillium wilt, characterized by slight yellowing of a few lower leaves. In contrast, the BS plants displayed more severe symptoms, including significant yellowing and leaf loss, with some plants eventually succumbing to the infection (Fig. 1a). The investigation of disease index and quantification of fungal biomass was conducted with reference to previous studies^23,24^. The disease index of BS was notably higher than that of BR at both 14 and 21 dpi (Fig. 1b). Assessing the cotton stem sections revealed browning in both transverse and longitudinal stem sections of BS plants after 14 days of *V. dahliae* inoculation, indicating a more severe condition compared to BR plants (Fig. 1c). Additionally, quantification of fungal biomass in the stems illustrated significantly higher accumulation in BS plants compared to BR plants at 14 and 21 dpi (Fig. 1d). Overall, these results convincingly demonstrated the significantly higher resistance of BR to *V. dahliae* than to BS.

### RNA Extraction, Library Preparation and Sequencing

Root samples from both the BR and BS genotypes were collected at 0, 12, 24 and 48 hours after *V. dahliae* inoculation separately. The samples were immediately frozen in liquid nitrogen. All samples were harvested from five cotton plants with three independent biological replicates. In total, 24 samples (2 genotypes × 4-time points × 3 biological replicates) were subjected to RNA-seq. For total RNA extraction, 0.1 g of the finely ground samples was accurately weighed and using the plant polysaccharide polyphenol total RNA extraction kit from TianGen Biotech Co., Ltd. (Beijing). From the extracted total RNA, 3 µg of qualified RNA was used for further processing. Specifically, mRNA was purified using magnetic beads with Okigo (dT) oligo for enrichment. Subsequently, the mRNA was fragmented, and first-strand cDNA synthesis was performed using a random primer with a six-base sequence. The second-strand cDNA synthesis was carried out by adding buffer, dNTPs, and DNA polymerase I. Subsequently, the double-stranded cDNA was purified using AMPure XP beads. To prepare the cDNA library, the purified double-stranded cDNA underwent end repair, A-tailing, and sequencing adapter ligation. Fragment size selection was performed using AMPure XP beads, and PCR amplification was conducted to obtain the cDNA library. The resulting library was subjected to RNA sequencing on the Illumina HiSeq 4000 platform, generating 150 bp paired-end reads. A total of 48 clean reads were obtained from the 24 cotton samples. The quality assessment of these raw RNA-seq reads (48 in total) was conducted using FastQC18 software, and the summarized results were generated using MultiQC software tool^25^. The raw sequencing data underwent data quality control with the fastp program, which involved the removal of sequences containing adapters, poly-N sequences, and low-quality data^26^.

### Differential expression genes (DEGs) identification and GO enrichment

The clean reads were aligned to the *Gossypium barbadense* L. (Hai7124) genome^27^ using hisat2 with default parameters^28^. FeatureCounts (Version 2.0.1) was used to calculate the read count of each gene, and gene expression levels were quantified using TPM^29^ (transcripts per kilobase of exon model per million mapped reads). Differentially expressed genes (DEGs) were identified using the DESeq R package (1.18.0)^30^ based on the criteria of |log2(fold-change)| ≥ 2 and an adjusted P value < 0.05. The gene Ontology (GO) enrichment analysis of DEGs was performed using the clusterProfiler^31^ R package, with a significance threshold set at an adjusted p-value < 0.05.

### Validation of DEGs by quantitative real-time PCR (qRT-PCR)

We selected 6 DEGs and designed gene specific primers online using the qPrimerDB website (https://biodb.swu.edu.cn/qprimerdb/), with amplified fragments ranging in length from 80 to 250 bp. The primers used in qRT-PCR were listed in Supplementary Table S1.

We performed the qRT-PCR experiment on Roche LightCycle 480II, using SYBR Green Mix. Additionally, *GbUBQ7* (DQ116441.1) was used as the internal reference gene and the relative expression level of the target gene is determined through Livak’s 2^−△△CT^ method^32^.

## Data Records

24 raw RNA-seq data reported in this paper have been deposited in the Genome Sequence Archive (Genomics, Proteomics & Bioinformatics 2021) in National Genomics Data Center (Nucleic Acids Res 2021), China National Center for Bioinformation/Beijing Institute of Genomics, Chinese Academy of Sciences (GSA: CRA012121)^33^ that are publicly accessible at https://ngdc.cncb.ac.cn/gsa/browse/CRA012121. Other data, such as the breakdown of each sample file on GSA, lists of raw gene counts, TPM of the 24 samples, the information of DEGs and results of GO enrichment analysis are also available at figshare^34^. (10.6084/m9.figshare.24097941.v2).

## Technical Validation

### Quality assessment of sequencing data

**Fig. 2:**
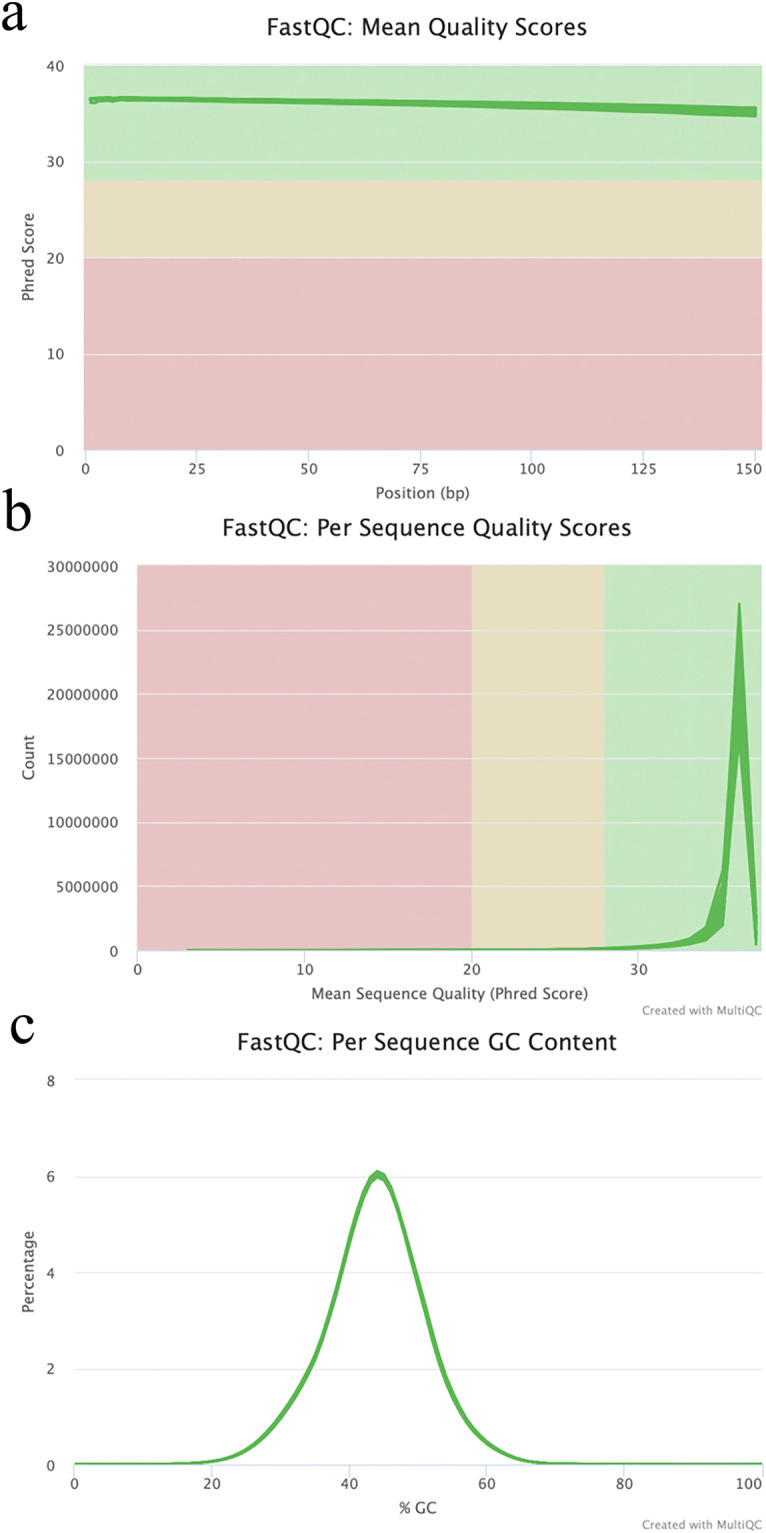
Evaluation of sequence quality in raw FASTQ data.The quality scores of all 48 raw RNA-seq reads were assessed using FastQC, and the results were compiled and summarized using MultiQC. (**a**) Displays the distribution of read counts for mean sequence quality. (**b**) Represents the distribution of mean quality scores. (**c**) Illustrates the per-sequence GC content.

The MultiQC analysis revealed that the quality scores of all sequences exceeded 30(Fig. 2a). Moreover, the per-sequence quality scores predominantly concentrated within the range of 35–40 (Fig. 2b), indicating a base error rate of less than 0.03%. The GC distribution of all sequences exhibited a normal distribution closely resembling the theoretical distribution (Fig. 2c), providing further evidence of the high quality of the reads. Throughout the data processing, an average of 5.9 million adapter sequences were filtered out from each sample, resulting in an average sample size of clean reads of 8.83 Gb (range 6.96–10.88 Gb). The percentage of clean reads was calculated as 99.4% (Table 1).

**Table 1:** RNA-seq data quality summary. All data above were counted for read1 + read2.

Sample_name	Accession	Clean_base (bp)	Raw reads	Clean reads	Effective rate(%)	Q20 (%)	Q30 (%)	GC content(%)
BR0A	CRR841041	7340324000	49509550	49228234	99.43	98.43	94.95	43.19
BR0B	CRR841042	7904142000	53177188	52872874	99.43	98.30	94.53	43.19
BR0C	CRR841043	10309525000	69464440	68978266	99.30	98.34	94.68	43.12
BR12A	CRR841044	6892841000	46425442	46129546	99.36	98.20	94.44	43.04
BR12B	CRR841045	9201922000	61987652	61601610	99.38	98.39	94.78	43.15
BR12C	CRR841046	8085703000	54372360	54072498	99.45	98.45	94.96	43.16
BR24A	CRR841047	7254628000	48880126	48576874	99.38	98.37	94.77	43.22
BR24B	CRR841048	8807664000	59285298	58956642	99.45	98.39	94.78	43.24
BR24C	CRR841049	7788918000	52521184	52213186	99.41	98.40	94.90	43.15
BR48A	CRR841050	7662713000	51597542	51274828	99.37	98.28	94.48	43.22
BR48B	CRR841051	9007112000	60665338	60296608	99.39	98.36	94.76	43.28
BR48C	CRR841052	7716154000	51952982	51636162	99.39	98.39	94.82	43.18
BS0A	CRR841053	7805688000	52561304	52246472	99.40	98.05	93.99	43.23
BS0B	CRR841054	9606783000	64661964	64266908	99.39	98.35	94.73	43.24
BS0C	CRR841055	9418788000	63448496	63078938	99.42	98.37	94.73	43.26
BS12A	CRR841056	8680751000	58390302	58060342	99.43	98.06	93.98	43.07
BS12B	CRR841057	10597593000	71386004	70992320	99.45	98.10	94.12	43.23
BS12C	CRR841058	10775798000	72558132	72116712	99.39	98.36	94.73	43.31
BS24A	CRR841059	8786492000	59146386	58814490	99.44	98.38	94.85	43.24
BS24B	CRR841060	9342497000	62871924	62502326	99.41	98.35	94.72	43.24
BS24C	CRR841061	10422598000	70139402	69753650	99.45	98.41	94.87	43.31
BS48A	CRR841062	8523364000	57428882	57030050	99.31	98.07	93.96	43.33
BS48B	CRR841063	8485533000	57163326	56799548	99.36	98.30	94.57	43.24
BS48C	CRR841064	9422100000	63381432	63029702	99.45	98.33	94.65	43.17

Raw bases: (Raw reads)* (sequence length). Clean bases: (Clean reads) * (sequence length). Effective Rate (%): (Clean reads/ Raw reads) *100%. Error rate: base error rate. Q20, Q30: (Base count value > 20 or 30)/(Total base count). GC content: (G and C base count)/(Total base count).

### Principal component analysis (PCA) and different basal transcriptome identification

**Fig. 3:**
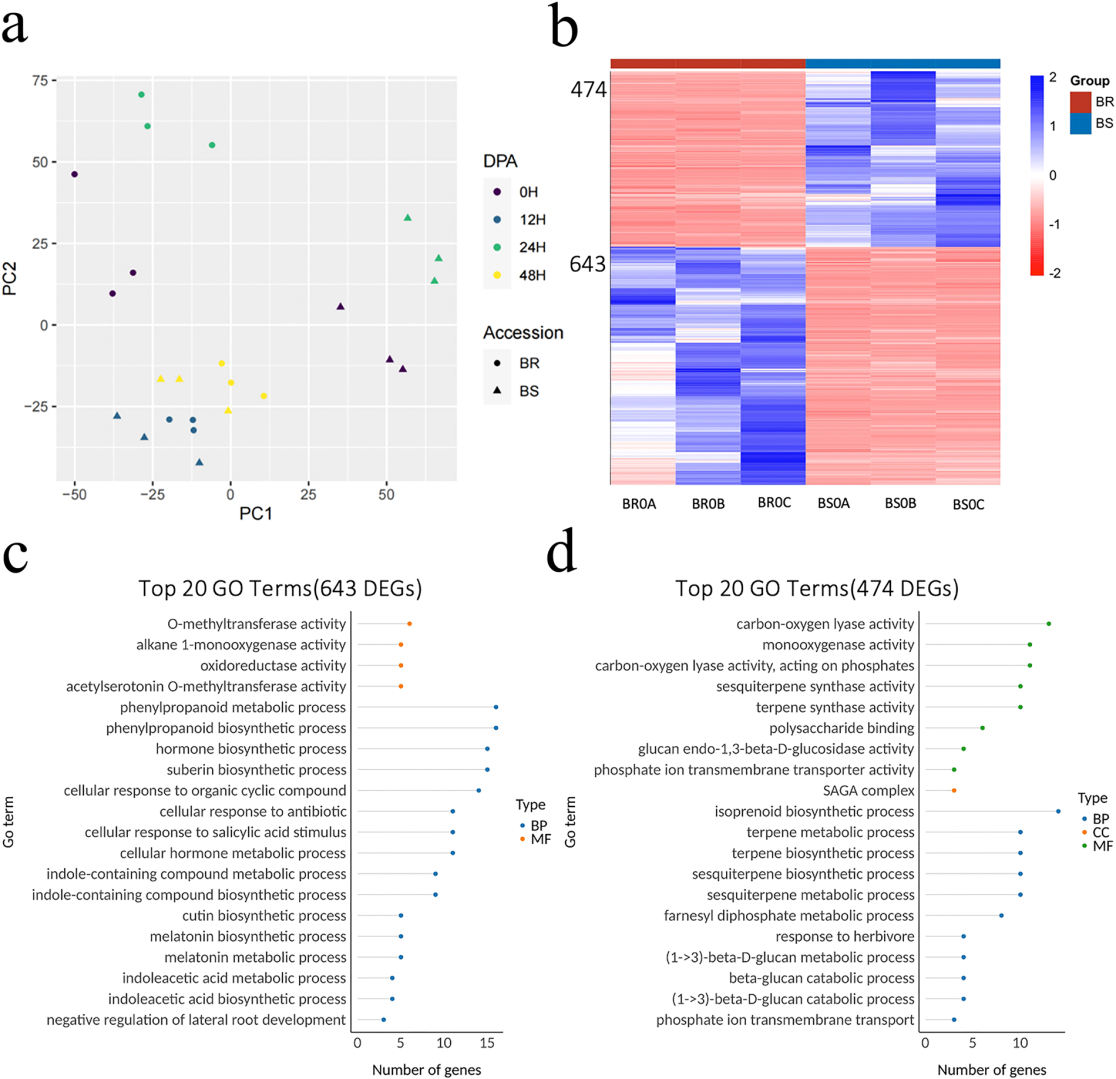
Basal transcriptomes of Xinhai 47 (BR), a resistant variety, and shidahaigan 1 (BS), a susceptible variety. (**a**) Principal component analysis (PCA) based on TPM values for 24 samples. (**b**) Heatmap showing the expression patterns of 1117 differentially expressed genes (DEGs) between BR and BS without *Verticillium dahliae* (*V. dahliae*) inoculation. (**c**) GO enrichment of highly expressed DEGs in uninfected BR plants compared to BS plants. (**d**) GO enrichment of highly expressed DEGs in uninfected BS plants compared to BR plants.

To evaluate the transcriptional differences between the BR and BS varieties, we performed principal component analysis (PCA) using the TPM values from 24 samples. The results demonstrated a clear distinction between the BR and BS varieties, regardless of *V. dahliae* inoculation (Fig. 3a, Figshare File 2^34^). Remarkably, even in the absence of *V. dahliae* inoculation, the BR and BS samples exhibited distinct separation based on PC1. This observation suggests a potential association between the transcriptional dissimilarities of the two varieties and their distinct levels of disease resistance. A total of 1117 DEGs were identified between the BR and BS varieties at 0 hours post-inoculation (hpi), prior to *V. dahliae* infection. Of these DEGs, 643 exhibited significantly higher expression levels in BR than in BS, whereas 474 displayed significantly lower expression levels in BR (Fig. 3b, Figshare File 3^34^). Among the top 20 GO terms in 643 DEGs, genes associated with physical barrier formation, such as *GbCYP86A1* (GB_A07G1113), *GbCER1* (GB_A10G0724) in suberin biosynthetic process were identified. Furthermore, a GO term related to Verticillium wilt resistance, which included cellular response to antibiotic pathways and melatonin biosynthetic process, was also significantly enriched in 643 DEGs (Fig. 3c, Figshare File 4^34^). The most enriched processes among the 474 DEGs with higher expression in BS were related to carbon-oxygen lyase activity and isoprenoid biosynthesis (Fig. 3d, Figshare File 5^34^).

### Analysis of differentially expressed genes (DEGs) involved in the response to *V. dahliae* inoculation

**Fig. 4:**
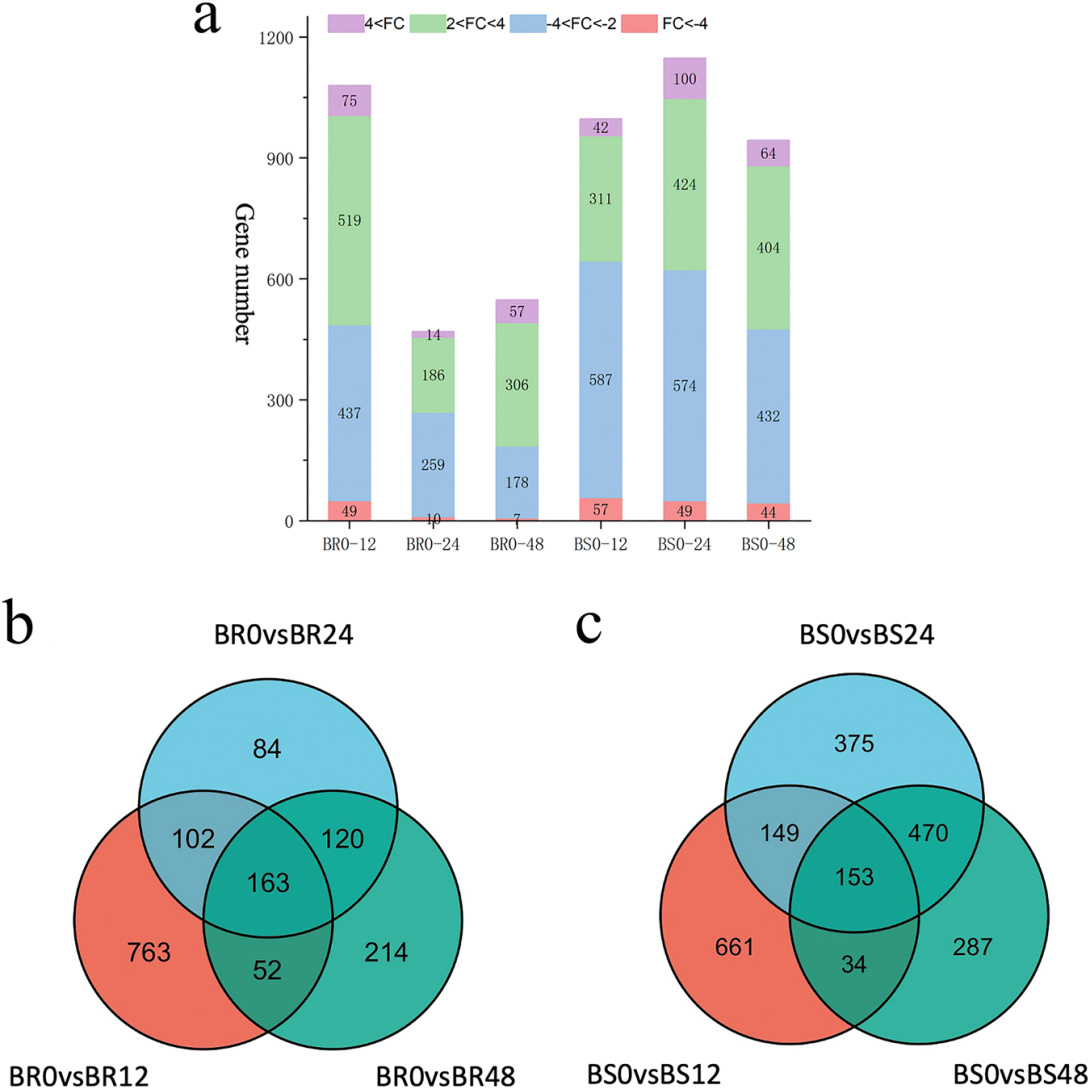
Identification of differentially expressed genes (DEGs) in Xinhai 47 (BR) and shidahaigan 1 (BS) at 12, 24, and 48 h after *Verticillium dahliae* infection. (**a**) The number of DEGs in BR and BS after *V. dahliae* infection at 12, 24, 48 hpi. (**b**) Venn diagram of DEGs at each time point in BR after inoculation with *V. dahliae*. (**c**) Venn diagram of DEGs at each time point in BS after inoculation with *V. dahliae*.

To investigate the molecular mechanisms underlying the resistance of different varieties of *G. barbadense* to *V. dahliae* infection, we compared the gene expression changes at 12, 24, and 48 hpi with an uninfected sample (0 hpi). A total of 1080, 469, 548 DEGs were identified at 12, 24, and 48 hpi in the BR variety respectively. Similarly, in the BS variety, we found 997, 1147, and 944 DEGs at the same time points (Fig. 4a, Figshare File 6^34^). Interestingly, BR demonstrated a high number of induced genes at all three time points, whereas BS displayed the opposite pattern. Analysis revealed 1498 nonredundant DEGs in BR and 2129 in BS, in response to *V. dahliae* at all three time points post-inoculation (Fig. 4b-c, Figshare File 7^34^).

**Fig. 5:**
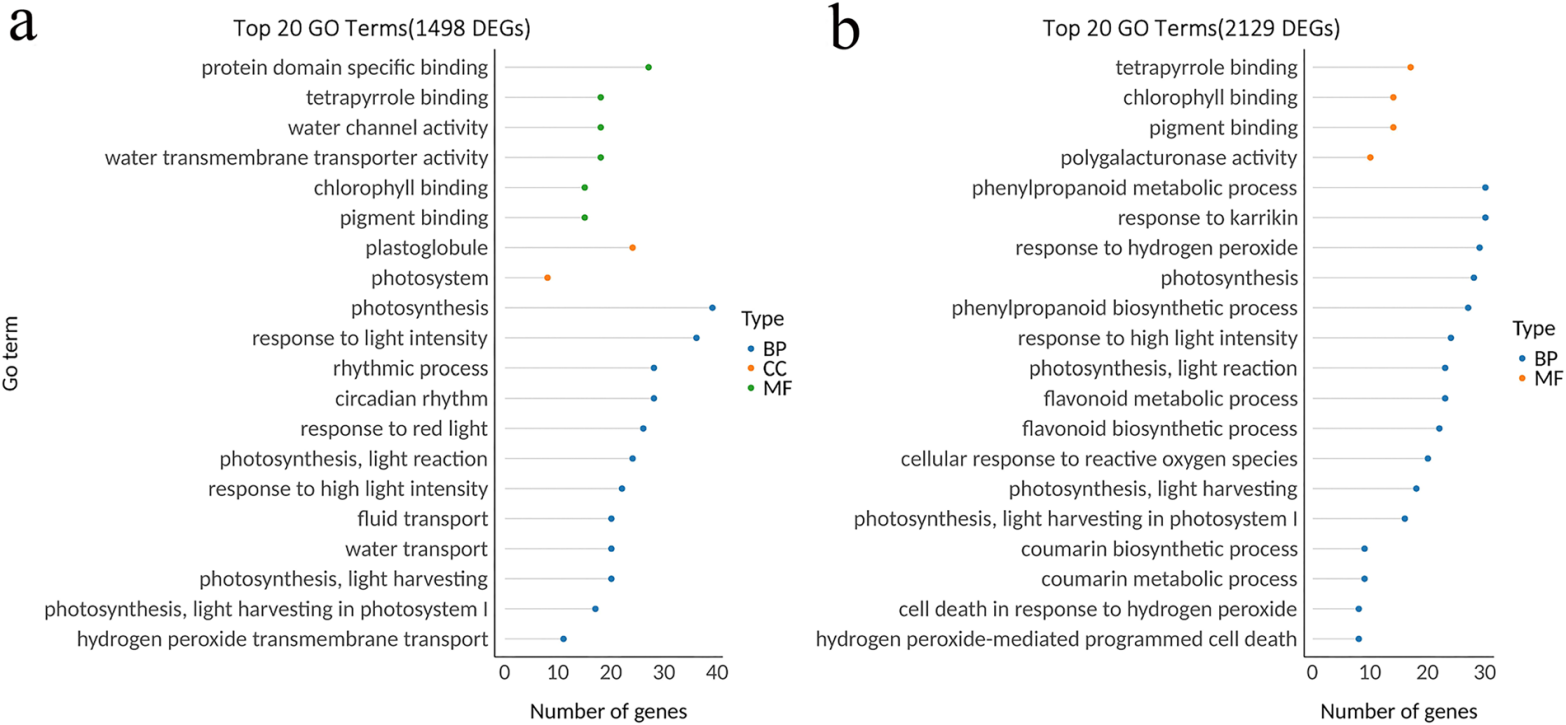
Gene Ontology (GO) analysis was performed on the 1498 and 2129 differentially expressed genes (DEGs) identified in the BR and BS varieties, respectively, after infection with *Verticillium dahliae* (*V. dahliae*). (**a**) GO analysis of DEGs in the BR variety following inoculation with *V. dahliae*. (**b**) GO analysis of DEGs in the BS variety following inoculation with *V. dahliae*.

Among the top 20 GO terms, 8 terms were shared between BR and BS. The most significantly enriched terms included photosynthesis, light reaction, and response to high light intensity (Fig. 5a, Figshare File 8^34^). Additionally, BS exhibited specific enrichment in phenylpropanoid metabolic process, response to hydrogen peroxide, and flavonoid metabolic process. These GO terms have previously been reported to play a role in cotton resistance to *V. dahliae* (Fig. 5b, Figshare File 9^34^). The findings suggest that BR displayed insensitivity to the pathogen, whereas BS exhibited a rapid activation of resistance-related transcriptome responses upon inoculation with *V. dahliae*.

### Validation results of RNA-seq data by qRT-PCR

**Fig. 6:**
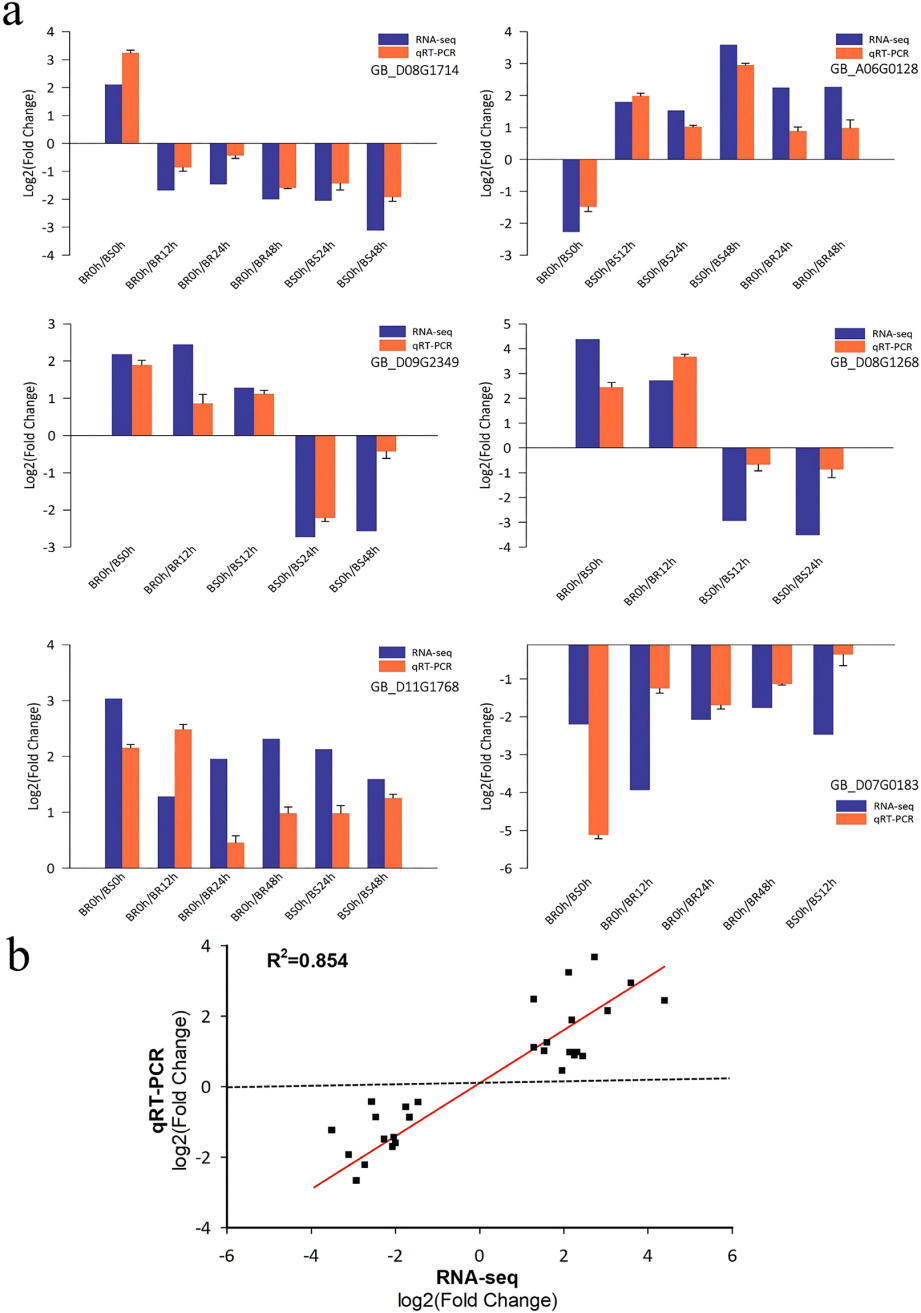
Validation of RNA-seq results by qRT-PCR analysis. (**a**) Fold change of six DEGs through qRT-PCR and RNA-seq. (**b**) Pearson correlation of the expression fold change of six DEGs between the RNA-seq and RT-qPCR assays.

Although the log2(fold-change) value from qRT-PCR did not precisely match that of RNA-seq, there was a strong positive correlation (R^2^ = 0.854) observed between the results of RNA-seq and qRT-PCR expression analyses, indicating the reliability of the RNA-seq results (Fig. 6).

In summary, we have presented a collection of high-quality transcriptome data illustrating the response of *G. barbadense* to *V. dahliae*. Our preliminary analysis indicates a potential association between the resistance of *G. barbadense* to *V. dahliae* and the presence of physical barriers. We believe that this transcriptome data offers valuable insights into the molecular mechanisms that underlie cotton’s resistance to *V. dahliae*, providing a novel approach for identifying key genes involved in such resistance.

### Supplementary information

Supplementary Table 1

## Data Availability

The following software used in the study was run with default parameters if not specifically stated in the Methods paragraph and no custom code was used for the purposes of this study. FastQC: https://www.bioinformatics.babraham.ac.uk/projects/fastqc (version 0.11.9). fastp: https://github.com/OpenGene/fastp(version 0.20.0). hisat2: https://daehwankimlab.github.io/hisat2/download(version 2.2.1). FeatureCounts: https://sourceforge.net/projects/subread(version 2.0.4). Differential analysis: R package DESeq 2 1.38.3. clusterProfile: R package clusterProfiler v4.6.2. Veen: https://hiplot.cn/basic/venn.
