# Supplementary material for: Transcriptomic datasets of Verticillium wilt resistant and non-resistant Gossypium barbadense varieties during pathogen inoculation
Source: Sci Data. 2024 Jan 2;11:11. doi: 10.1038/s41597-023-02852-2 (PMC10762110; doi:10.1038/s41597-023-02852-2)
Supplement: Supplementary file 1 — Supplementary Table 1 [file 41597_2023_2852_MOESM1_ESM.pdf]

Supplementary Information

Transcriptomic datasets of Verticillium wilt resistant and non-resistant *Gossypium barbadense* varieties during pathogen inoculation

Xianpeng Xiong [1,5], Cong Sun [2,5], Bin Chen [3], Jie Sun[3], Cong Fei [4], Fei Xue [3]

[1] Shenzhen Branch, Guangdong Laboratory of Lingnan Modern Agriculture, Genome Analysis Laboratory of the Ministry of Agriculture and Rural Affairs, Agricultural Genomics Institute at Shenzhen, Chinese Academy of Agricultural Sciences, Shenzhen, China 518120.
[2] State Key Laboratory of Cotton Biology, Institute of Cotton Research, Chinese Academy of Agricultural Sciences, Anyang 455000, China.
[3] Key Laboratory of Oasis Eco-Agriculture, College of Agriculture, Shihezi University, Shihezi 832000, China.
[4] Department of Life Sciences, Yuncheng University, Yuncheng 044000, China
[5] These authors contributed equally.
corresponding author(s): Fei Xue and Cong Fei

Supplementary Information Index

Supplementary Table S1. The primers used in this study. **Page 2**

Supplementary Table S1. The primers used in this study.

Primer name	Sequences (5'-3')
GB_D08G1714F	ACTTCTTTTGGTGCCATGGC
GB_D08G1714R	ATCGACGTGGAATGCAATGG
GB_A06G0128F	TCGGCGGCATGAAAATCTTC
GB_A06G0128R	CGGCATTTGTTCCATTGCAC
GB_D09G2349F	TCAAGGTCCGGTCAGTTCATG
GB_D09G2349R	AAAGCCAAGGCAACAAGTCC
GB_D08G1268F	AAAGACAACCAAGGCACAGC
GB_D08G1268R	GTCCAGCATTTGACGCATTG
GB_D11G1768F	AAAGCCTCCGCAATCATTCC
GB_D11G1768R	TTGATCGTGGATGCCGAAAC
GB_D07G0183F	TTGCACATTGGCACTTCGTC
GB_D07G0183R	TTGGCACGATCCGACAATTC
GbUBQ7F	GAAGGCATTCCACCTGACCAAC
GbUBQ7R	CTTGACCTTCTTCTTCTTGTGCTTG
